# Cutaneous filarioid nematodes of dogs in the United States: Are they emerging, neglected, or underdiagnosed parasites?

**DOI:** 10.3389/fvets.2023.1128611

**Published:** 2023-02-23

**Authors:** Jeff Gruntmeir, Maureen Kelly, Rafael Antonio Nascimento Ramos, Guilherme Gomes Verocai

**Affiliations:** ^1^Emerging Pathogens Institute, University of Florida, Gainesville, FL, United States; ^2^Department of Infectious Diseases and Immunology, College of Veterinary Medicine, University of Florida, Gainesville, FL, United States; ^3^Southeastern Center of Excellence in Vector Borne Diseases, Gainesville, FL, United States; ^4^Department of Veterinary Pathobiology, School of Veterinary Medicine and Biological Sciences, Texas A&M University, College Station, TX, United States; ^5^Laboratory of Parasitology, Federal University of the Agreste of Pernambuco, Garanhuns, PE, Brazil

**Keywords:** *Onchocerca*, *Cercopithifilaria*, *Dirofilaria*, diagnosis, vector-borne diseases, zoonosis

## Abstract

Filarioid nematodes, which are vector-borne parasites of cosmopolitan distribution, of dogs are medically important. They are represented by species in which microfilariae were found to be circulating in the bloodstream (e.g., *Dirofilaria* sp., *Acanthocheilonema* sp., and *Brugia* sp.) or skin-dwelling (e.g., *Cercopithifilaria* sp. and *Onchocerca* sp.). Those species whose microfilariae are detected in blood have been extensively studied, especially *Dirofilaria immitis*, due to their clinical importance. In recent decades, there has been an increased interest by the scientific community in filarioid nematodes whose microfilariae are detected in the skin because of the zoonotic aspect of *Onchocerca lupi*. In the United States (US), although *D. immitis* has been considered the main filarioid infecting dogs, the intense animal movement and global canine filarioid diversity may indicate that the likely presence of cutaneous filarioid nematodes is more common than previously expected. Hence, a question remains: Are these canine filarioid nematodes emerging, neglected, or simply underdiagnosed in the US? In this review, we provide an overview of pertinent information that briefly summarizes the biology of the different canine filarioid nematode species, clinical signs associated with infections, and currently available diagnostic tools using molecular and microscopy-based methods and highlight knowledge gaps where research and surveillance efforts remain necessary. The data herein presented serve as an alert to the scientific community about the importance of filarioid nematodes infecting dogs other than *D. immitis*. Additionally, the zoonotic potential of several filarioid species reinforces the necessity of a proper diagnosis and the need for broader surveillance to understand their diversity and distribution, to highlight the potential introduction of certain species, and mitigate their establishment in the country and new animal and human cases.

## 1. Introduction

Parasitic nematodes within the family Onchocercidae (order Spirurida, superfamily Filarioidea) are of significant medical and veterinary importance globally. Species within all genera are transmitted to the vertebrate definitive host *via* blood-feeding arthropod intermediate hosts including mosquitoes, flies, fleas, lice, and ticks (1–4). A recent study that revisited the phylogenetic relationships among Onchocercidae proposed changes in the previously accepted subfamily-level taxonomic classification (2, 5). The family is composed of 5 distinct clades, namely, ONC-1 to ONC-5. Species within two genera within the ONC3 clade, *Dirofilaria* and *Onchocerca*; two genera within ONC4, *Acanthocheilonema* and *Cercopithifilaria*; and one genus in ONC5, *Brugia*, are known to infect domestic dogs (2, 6). From a global perspective, at least 14 onchocercid species are known to infect dogs, including those whose microfilariae are primarily found in the blood, *Acanthocheilonema reconditum, Acanthocheilonema dracunculoides, Acanthocheilonema* sp. “Ladhki genotype,” *Brugia ceylonensis, Brugia malayi, Brugia pahangi, Brugia patei, Dirofilaria* sp. “hongkongensis genotype,” *Dirofilaria immitis*, and *Dirofilaria repens*, and those with microfilariae are detected in the skin, *Cercopithifilaria bainae, Cercopithifilaria grassi, Cercopithifilaria* sp. II, and *Onchocerca lupi* (7–15). Adult nematodes of most species responsible for canine filariosis are found primarily in the subcutaneous and connective tissues and the lymphatic system. Some are also exceptionally adapted to the pulmonary arteries and associated vasculature, as in the case of *D. immitis*.

Global animal movement and pet travel within and between countries and over large geographic distances have been recognized as a global veterinary and public health concern, causing the dissemination of vectors and potentially zoonotic infections (parasitic, viral, and bacterial) into new areas (16). Dog importation into the United States (US) was estimated at ~1.06 million in 2019, according to a USDA report with ~36% of those imports coming across land borders with Canada (12%) and Mexico (~24%) (17). Following decades of animal movement, importation and considering the global canine filarioid diversity, where are the manifestations of clinical cases in shelter and pet populations, as well as in humans? Are these canine filarioid nematode species emerging, neglected, or simply underdiagnosed in the US?

Active research, scientific awareness, and veterinary medical knowledge of filarioid nematode diversity infecting domestic and wild carnivores in some areas of the Old World, particularly Europe, are much further established than those of the New World, including North, Central, and South America. Among several factors contributing to the lack of knowledge for filarioid species in domestic dogs in the US, is the repeated assumption that heartworm is the only filarioid of clinical importance across the New World. A result of this assumption and an overreliance on heartworm antigen testing was an established perception that testing for microfilariae detection had limited clinical value and was not necessary (18–20). However, in the absence of routine microfilariae testing or an awareness of other canine filarioid nematodes, frontline veterinarians in the US are unlikely to find or suspect any filarioid species other than heartworm in their canine patients, and to a lesser extent, *A. reconditum*, except for the chance of an incidental finding. A recent example that should cause alarm is that in only one of the 10 US surveys reporting microfilariae testing in dogs (21–27) conducted since 1989 (28, 29), ~2% of dogs and cats in a south Florida shelter had circulating microfilariae morphologically identified as *D. repens* by veterinary parasitologists performing modified Knott's testing and molecularly confirmed by sequencing, although these sequences were not made available in any repository (23). This finding in a dog and a cat in the same US shelter strongly suggests that local transmission of this zoonotic parasite may be occurring in our highest-risk populations, in an area supporting competent mosquito vectors and where shelter animal evacuations are frequent due to hurricanes. While the origin and detailed travel history of these animals were unknown, it is likely that additional infections with *D. repens* and other imported filarioid nematodes are present in US shelter, rescue, and pet populations. Previous reports described a dog in New York that was microfilaremic for *D. repens*, and blood samples from six animals were submitted to a reference lab for microfilariae identification after the heartworm antigen test result was negative (30). The dog had originated in the Czech Republic and moved through the Netherlands and Canada before arriving in New York (30). Animals that are microfilaremic from non-endemic filarioids pose a risk of initiating cycles of autochthonous transmission within the US or non-endemic areas, being detectable only by microfilaria testing or clinical presentation. These examples underscore the need for microfilariae testing and broad awareness of possible emerging parasites among veterinary researchers, veterinarians, and public health entities. Despite advances in molecular diagnostic tools capable of detecting and differentiating filarioid nematodes to species-level, no published *D. immitis* or blood microfilariae prevalence or research surveys have applied molecular tools alone or in combination with morphological identifications for screening in the US prior to 2020 for sheltered dogs (23–25) and wild canids (31), or prior to 2022 for pet dogs (26, 27, 32).

Historically, *A. reconditum* and *D. immitis* have been recognized as endemic in the US, with *O. lupi* and *C. bainae* being unequivocally recognized only since 2011 and 2019, respectively (33–35). Most global research and surveillance efforts for filarioid nematodes of dogs have focused on the detection and differentiation of *D. immitis*, due to its major veterinary importance, or *D. repens* and multiple *Brugia* species of zoonotic importance in Europe, south and southeastern Asia, and Africa, where dogs, cats, and other carnivores may serve as reservoirs of infection (26). While generally considered neglected, the subcutaneous canine filarioid nematodes with dermal microfilariae such as *O. lupi* did not gain research attention until it was attributed to human cases in areas where this parasite is endemic, in particular within the southwestern US (36–39). In addition to *O. lupi*, research on the presence of *Cercopithifilaria* in the US has only recently been increasing (35, 40, 41) following the plethora of published research and case reports, originating from Europe, primarily Italy, and the broader Mediterranean region, which has highlighted possible differentials for clinical presentations attributed to these neglected nematodes (42–44).

Awareness of potentially imported or emerging parasites, and particularly canine filarioid nematodes, is lacking among shelter and general practice veterinarians. Active efforts to inform veterinarians of emerging parasitic infections are needed through conferences and continuing education opportunities. Additionally, proactive surveillance is needed by veterinary parasitologists and ideally, medical entomologists or public health offices involved in vector-borne disease (VBD) surveillance of mosquito, biting fly, and tick vectors. It is essential that all relevant case reports of emerging filarioid nematodes from diagnostic and reference laboratories were published or otherwise formally communicated among peers. This is particularly important for species with known zoonotic potential, even without the need for official communication with or reporting to state or federal authorities. The purpose of this review article is to provide an overview of pertinent information that briefly summarizes the biology of the different canine filarioid nematode species, clinical signs associated with infections, and currently available diagnostic tools using molecular and microscopy-based methods, as well as to highlight knowledge gaps where research and surveillance efforts remain necessary.

## 2. Subcutaneous filarioid nematodes with dermal microfilaria

### 2.1. *Cercopithifilaria* spp.

#### 2.1.1. Biology

The genus *Cercopithifilaria* (Filarioidea, Onchocercidae) is composed of 28 species infecting a variety of mammal definitive hosts, including primates, carnivores, and ungulates (45). In domestic dogs, three species have been described or reported, namely, *C. grassi, C. bainae*, and a distinct species that has not been formally described, *Cercopithifilaria* sp. II sensu (42). Adults of *Cercopithifilaria* species are found in the subcutaneous tissues of dogs (46, 47). The first description of *Cercopithifilaria* infecting dogs dates from 1907 in Italy when Noè detected microfilariae in skin samples (48). Initially, this species was named *Filaria grassii* and later transferred to the genus *Cercopithifilaria* (49). *Cercopithifilaria bainae* was originally described based on specimens isolated from dogs in Brazil (50), and it has recently been redescribed based on material from dogs in Italy using integrated classical and molecular methods (51).

Although known for a long time, the biology of *Cercopithifilaria* species has been poorly investigated (52, 53). Knowledge related to the development of this parasite in vertebrate hosts is almost non-existent. Microfilariae are unevenly distributed on the dog's body but seem to have a predilection for the head, the ears, and the neck, which coincidentally are the preferred sites of attachment of the brown dog tick, *Riphicephalus sanguineus sensu lato* (s.l.), the only proven intermediate host for *C. bainae* (54). Despite the fact that *C. bainae* microfilariae occur in the skin, three previous studies have reported the occurrence of its microfilariae in the bloodstream (55–57), a possible introduction during blood collection by venipuncture. While unusual, this finding is important to consider during morphological or molecular diagnosis of filarioid species with blood microfilariae.

The only unequivocally proven intermediate host for *C. bainae* is the brown dog tick, *R. sanguineus* (s.l.) (52). A prepatent period of <6 months is suggested (54). It was previously demonstrated experimentally that infection is acquired during the nymphal blood meal and passed by transstadial transmission to adults as the L3 infective stage ~30 days after nymph detachment (52, 53). The most recent update on the biological development in the tick vector for *C. bainae* originated from researchers who conducted retrieval in dogs from Italy (46, 52). In this study, larvae inside ticks were classified into four different developing types based on their morphometrical features, with sizes ranging from 191.4 μm (±9.1; DL1—developing first stage) to 1,707 μm (±70.5; L3–infective third stage larvae) (52). The potential role of other tick species such as *Ixodes ricinus* was also assessed but without success (53). More recently, the DNA of *C. bainae* was detected in *Rhipicephalus haemaphysaloides* from India, which suggest that ticks from different species or genera may be putative vectors for this nematode (47). However, it is important to highlight that further studies are needed to prove the potential vectorial role of this later species. Despite limited data regarding the adult *C. bainae* nematodes, these have been found in the subcutaneous tissues of the trunk and forelimbs and less frequently in the perirenal adipose tissue (51).

Various studies suggested that *R. sanguineus* (s.l.) may comprise a species complex (58, 59). In the US, the presence of two *R. sanguineus* lineages has been molecularly recognized, a “temperate lineage” and a “tropical lineage,” both with wide and overlapping distributions across the country (60). Thus, far, the DNA of *C. bainae* has been obtained only from *R. sanguineus* specimens belonging to the “temperate lineage” (40). It remains to be confirmed whether “tropical lineage” brown dog ticks are also capable of transmitting *C. bainae* and whether there is any difference in vector competence between lineages.

#### 2.1.2. Geographic distribution

The geographic distribution of *C. bainae* is vast and overlaps with that of *R. sanguineus* (s.l.) (52, 53, 61). Overall, *Cercopithifilaria* species infecting dogs are widespread, being reported on different continents. In the US, there are only three publications reporting the occurrence of *C. bainae* in dogs or ticks (Table 1). The first report of *C. bainae* in the US was from a dog from Florida (35), and the second was from shelter dogs and *R. sanguineus* (s.l.) ticks from Oklahoma (40). Subsequently, an extensive study that molecularly screened *R. sanguineus* (s.l.) ticks collected from dogs detected DNA of *C. bainae* in larval, nymphal, and adult brown dog ticks from 11 different American states, including Florida and Oklahoma (41).

**Table 1:** Reports of *Cercopithifilaria bainae* and *Onchocerca lupi* infection in dogs and vectors in the US.

***Cercopithifilaria bainae*** **(vertebrate host—dog)**				
**State**	**Cases (** ***n*** **)**	**Parasite stages**	**Type of diagnosis**	**References**
Florida	1	Microfilariae	Histopathology, morphology, and molecular	(35)
Oklahoma	6	Microfilariae	Microscopy and molecular	(40)
***Cercopithifilaria bainae*** **(vector—tick)**				
Oklahoma	3	Developing stages	Molecular	(40)
Arkansas	3	Developing stages	Molecular	(41)
Arizona	16	Developing stages	Molecular	
California	2	Developing stages	Molecular	
Colorado	6	Developing stages	Molecular	
Florida	4	Developing stages	Molecular	
Kentucky	1	Developing stages	Molecular	
New Mexico	7	Developing stages	Molecular	
Oklahoma	2	Developing stages	Molecular	
Texas	35	Developing stages	Molecular	
Utah	3	Developing stages	Molecular	
Wisconsin	1	Developing stages	Molecular	
***Onchocerca lupi*** **(vertebrate host—dog)**				
Arizona	1	Adult (gravid female)	Morphology	(62)
	1	NA	Histopathology and molecular	(63)
California	1	Adult (gravid female and male)	Histopathology and morphology	(64)
	1	Adult (female and male)	Histopathology and morphology	(65)
	1	Adult (gravid female)	Histopathology and morphology	(62)
	2	Adult (gravid female and male)	Histopathology and morphology	(66)
	1	Adult (gravid female)	Histopathology and molecular	(67)
	3	Adult (fragment of nematode)	Morphology and molecular	(68)
Colorado	1	Adult (gravid female)	Histopathology and molecular	(67)
	2	Adult	Morphology and molecular	(69)
	2	Adult	Morphology and molecular	(70)
	2	NA	Molecular	(31)
Florida	1	Adult	Morphology and molecular	(69)
	1	Adult nematode	Morphology and molecular	(70)
Minnesota	1	Adult	Morphology and molecular	(69)
	1	Adult	Morphology and molecular	(70)
Nevada	1	Adult (female)	Histopathology and molecular	(67)
New Mexico	4	Adult	Morphology and molecular	(69)
	4	Adult	Morphology and molecular	(70)
	16	Adult	Histopathology and molecular	(71)
	21	NA	Molecular	(31)
New York	1	Adult	Histopathology, morphology, and molecular	(72)
Texas	1	Adult (gravid female)	Histopathology and molecular	(73)
Utah	1	Adult (gravid female)	Histopathology and morphology	(65)
	1	Adult (gravid female)	Histopathology and molecular	(67)
***Onchocerca lupi*** **(putative vector*****—Simulium tribulatum*****)**				
California	6	NA	Molecular	(68)

#### 2.1.3. Pathogenicity, clinical signs, and diagnosis

The pathogenicity of *Cercopithifilaria* species infecting dogs has not been fully elucidated. Nonetheless, there are few reports in which *C. bainae* has been suggested as cause of dermatitis and polyarthritis in dogs from Europe (54, 74), dermatitis in the US (35), and more recently to the presence of a giant cutaneous cyst in a dog from Brazil (75). First, skin alterations recorded in a dog in Italy were characterized by a diffuse, erythematous, papular dermatitis (54). During the histological examination, the presence of neutrophils, eosinophils, and lymphocytes was observed in association with microfilariae (54). Afterwards, a 7-year-old dog from the same country presented a history of reluctance to move, lethargy, and lameness. With the exclusion of other potential pathogens, the presence of *C. bainae* in the synovial fluid was considered an aberrant localization that most likely triggered an inflammatory reaction (74). It is believed that this reaction is similar to that induced by *Cercopithifilaria johnstoni* in the perivascular connective tissues of infected rodent definitive hosts (76).

In the US, a dog from Florida with no domestic or international travel history presented with an annular erythematous plaque on the head and ulcers on the medial canthi that were unresponsive to antibiotic treatment (35). The histopathological evaluation revealed an eosinophilic to lymphohistiocytic perivascular dermatitis associated with *C. bainae* microfilariae. The dog was treated with a commercial spot-on formulation containing imidacloprid and moxidectin, and clinical resolution was achieved (35). In Brazil, a recent report describes a 9-year-old male mixed-breed dog who presented with a mass in the lumbosacral region. At the cytological examination, moderate lymphocyte cellularity, foamy macrophages, and erythrophagocytosis were observed. The presence of numerous microfilariae was also detected in the cyst fluid, subsequently identified as *C. bainae* (75). Although speculative, the large number of microfilariae in the cyst fluid and the absence of coinfection with other pathogens may suggest a potential involvement of this filarioid species. For the first-time subcutaneous effusion and *C. bainae* microfilariae were noticed together, suggesting an intense immune response against the parasite, with probably fluid sequestration or lymphatic obstruction (75).

The diagnosis of *Cercopithifilaria* spp. infection in dogs is discussed below, along with that of *O. lupi*.

### 2.2. *Onchocerca lupi*

#### 2.2.1. Biology

The genus *Onchocerca* comprises more than 30 valid species, the vast majority infecting wild and domestic ungulates, with a few exceptions such as *O. lupi*, which infects carnivores, and *O. volvulus*, which infects humans (6). Many *Onchocerca* species associated with animal hosts have been shown to be zoonotic, including *O. lupi* and other species associated with ruminants and suid hosts.

Similar to all filarioid nematodes, *O. lupi* has an indirect life cycle, and dogs seem to be its most common definitive host (77). However, the species was originally described from a wolf (78), and there have been many reports of infection in cats (34, 79, 80), coyotes (*Canis latrans*) (81), and humans (38, 39, 82, 83). Usually, the adult worm develops within the connective tissues, particularly the ocular conjunctiva, and microfilariae are released near these regions, eventually being detected in the skin of the definitive host. Microfilariae must be ingested by an arthropod intermediate host(s), in which it will develop to the infective third-stage larvae.

The vector or vectors of *O. lupi* remain unknown, but based on the biology of other *Onchocerca* species, it is believed that black flies (Diptera, Simuliidae) and/or biting midges (Diptera, Ceratopogonidae) may act as intermediate hosts. Even if not biologically confirmed, this hypothesis is reinforced by the retrieval of *O. lupi* DNA in *Simulium tribulatum* from southern California (68), a species vastly distributed across North America (84). Despite not being featured in any peer-reviewed publication, there have been *O. lupi* sequences isolated from the heads and bodies of *Simulium griseum* from New Mexico, further supporting the putative role of black flies as suitable intermediate hosts. Several studies in Europe failed in the attempt to confirm the role of black flies and other biting flies as biological vectors of *O. lupi*, likely due to poor sampling (77, 85, 86). It is known that this parasite species presents a wide geographic distribution; thus, the possibility of multiple arthropod species acting as vectors (e.g., as occurs with *Onchocerca volvulus*, the agent of river blindness in humans in Africa and Latin America) cannot be ruled out (87). Biting midges (Ceratopogonidae) should also be further investigated as possible vectors since they are known to transmit multiple *Onchocerca* species in the US including the equine *O. cervicalis*, and the bovine *O. gutturosa* (88–91) as well as others worldwide (1, 92). Although a wide range of *Culicoides* species are present in *O. lupi* endemic areas of the southwestern US, many of which are documented to feed primarily on humans (93), two subspecies within the *Culicoides variipennis* complex, *Culicoides v. sonorensis* and *Culidoides v. occidentalis* (94), are known to feed on both dogs and humans (93), and *Culidoides v. variipennis* has been shown to naturally and experimentally transmit *O. cervicalis* to horses in the US (95, 96). Additionally, the apparent distribution of *Culidoides v. occidentalis* (94) encompasses the known endemic areas in the southwestern US coinciding with human, companion animal, and coyote cases (70, 81). The combined morphological and phylogenetic evidence suggest that *O. lupi* has a recent evolution history and clusters with human and bovine *Onchocerca* spp. (6, 82, 97), and considering that the known bovine-derived *Onchocerca* spp. (*O. ochengi, O. gutturosa*, and *O. gibsoni*) are all vectored by species of *Culicoides* (88–91, 98), it should provide further support for examination of mammalophilic *Culicoides* spp. in natural populations or experimentally. Additionally, in the Amazon of South America, presumed infective larvae of an *Onchocerca* species most closely related to *O. lupi* based on phylogenetic analysis using the COI gene were found in the labium of the proboscis and head of two sandflies (Psychodidae, Phlebotominae), suggesting this group of biting flies should also be investigated in relevant *O. lupi* endemic regions (99).

Despite the data presented in clinical reports of onchocercosis in dogs, information about the biological aspects of this nematode in this vertebrate host remains scarce. For instance, the pre-patent period and patency of *O. lupi* are unknown. *Onchocerca lupi* microfilariae have been found in higher abundance on the skin of the ears and nose, followed by the forehead and interscapular area of naturally infected dogs (100). It is possible that the choice of anatomic location(s) for skin sampling may impact the prevalence estimate for *O. lupi* in epidemiological studies. A single study has shown that *O. lupi* microfilariae were detected in higher abundance in skin samples collected in the afternoon, followed by night and morning (100), likely coinciding with periods of higher activity of suitable vectors. More recently, it has been reported that large black dogs presented a higher risk to develop onchocercosis compared with similar sized brown or white ones (101). It has been well-established that various hematophagous dipteran species, including black flies, are more attracted to darker colors and therefore tend to feed on darker animals (101–103). Regarding a dog's body size, it may be hypothesized that a larger dog likely emits more CO_2_ than smaller ones, making it more attractive to host-seeking dipterans. On the contrary, there could be various additional factors influencing these variables, including time spent outdoors.

#### 2.2.2. Geographic distribution

After decades from the original description of *O. lupi*, a few cases of *Onchocerca* infection were reported in dogs from the US (62, 64). Initially, the identity of the etiological agent was putatively attributed to a species other than *O. lupi* (e.g., *Onchocerca lienalis*), as there were no records for the species from the New World. Later, four cases of canine ocular onchocercosis were reported in Hungary, and the morphologically informative features of this nematode allowed species-level confirmation of the agent as *O. lupi* (104). After the confirmation of *O. lupi* infection using integrated morphological and molecular methods in dogs, cats, and humans in the US (34, 36, 67), it has been assumed that previous cases of ocular infection in dogs and humans could be attributed to this species. For instance, a revisited study demonstrated that two filarial ocular human cases in Turkey (originally identified as *D. repens*) and Tunisia (originally identified as *D. immitis*) were attributed to *O. lupi* (105). The interest of researchers, veterinarians, and public health professionals has substantially grown in North America and the Old World.

In more recent years, there have been multiple *O. lupi* reports in companion animals, especially in dogs and less frequently in cats, worldwide, including in North America (34, 64, 66, 67, 70, 73, 106). Coincidentally, the highest incidence of clinical *O. lupi* infections in dogs is in the southwestern US, in special New Mexico and Arizona (71, 107), where several human cases have been reported (38, 39). In addition, coyotes, which are vastly distributed across North America, were demonstrated as *O. lupi* hosts and may act as wild reservoirs (81). There has been strong evidence that this filarioid may be undergoing range expansion into new areas, perhaps promoted by the movement of dogs, both domestically and internationally. For instance, two adoption-mediated cases of canine ocular onchocercosis have been reported from Canada (106), with animals coming from endemic areas of the US. Similarly, in the US, there have been increasing reports of ocular onchocercosis in dogs from areas currently considered non-endemic. More recently, an autochthonous case has been reported from south Texas, expanding its known endemic distribution in North America (73). Since this publication, the authors have confirmed additional *O. lupi* cases in dogs from Texas; nevertheless, its prevalence and distribution across the state remain unknown. Table 1 shows all published reports of *O. lupi* infection in dogs in the US; however, the authors have confirmed *via* classical and molecular methods dozens of additional cases.

#### 2.2.3. Pathogenicity, clinical signs, and diagnosis

In dogs, *O. lupi* is generally responsible for the ocular disease (69, 71, 73), which may be characterized by acute or chronic ocular signs (100). The pathogenicity of this parasite is mainly related to the presence of adult worms in the extraocular tissues, which is usually accompanied by the presence of a mass or swelling in the ocular region (101). In most cases, the swelling occurs due to the presence of epithelioid macrophages and multinucleated giant cells (71), as a result of a host response against the parasite. Several clinical signs may be observed, such as lacrimation, conjunctivitis, exophthalmia, retinal detachment, and the presence of granulomatous nodules, which may lead to blindness (70, 82).

Clinical signs associated with ocular disease may be present in one or both eyes (71); however, data from epidemiological surveys suggest that many animals may remain asymptomatic and would likely go undiagnosed. In addition to the classical ocular presentation of *O. lupi* infection, a few cases of aberrant localization of *O. lupi* have been reported, including the respiratory system (108, 109). For instance, in Portugal, in a dog with a history of acute and severe dyspnea and cyanosis, adult nematodes were detected in the laryngeal region (109). Similarly, in Greece, in an animal presenting coughing, difficulty in breathing, and edema near the laryngeal area, microfilariae were detected in buccal lymph nodes (110). More recently, a case of extensive aberrant localization of *O. lupi* was described in the US, with nematodes or degenerate nematodes detected in different anatomical regions (e.g., episclera, trachea, subcutis around the nares, external ear canals, parietal pleura, pericardium, and laryngeal cartilage) (63). While the pathogenicity of *O. lupi* is known to be associated with adult nematodes, it remains unclear whether the presence of its skin-dwelling microfilaria may cause dermatitis or any dermatological condition, similar to *O. volvulus* (111).

Filarioid nematodes whose microfilariae are skin-dwelling have been neglected in veterinary medicine for a long time. Undoubtedly, infections by *Onchocerca* and *Cercopithifilaria* are less commonly diagnosed than those by filarioid nematodes whose microfilariae are circulating, such as *D. immitis, D. repens, A. reconditum*, and *A. dracunculoides* (112). In this context, it is paramount to increase the awareness of veterinarians and researchers about their veterinary and public health relevance and better inform them on how to diagnose these neglected parasites, which often require integrated classical, microscopy-based methods and molecular tools.

**Figure 1:**
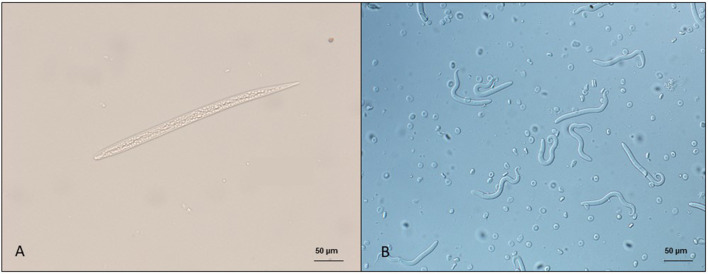
Microfilariae of dermal filarioids in dogs. **(A)** *Cercopithifilaria bainae* microfilaria isolated from a skin sample; scale bar: 50 μm. **(B)** *Onchocerca lupi* microfilariae recovered from the uterus of an adult nematode; scale bar: 50 μm.

The diagnosis of skin-dwelling filarioid infections can be achieved by morphometric analysis of microfilariae isolated from a skin sample *via* microscopy (Figures 1A, B). Briefly, skin samples may be collected using a disposable scalpel or a biopsy punch (from 3 to 8 mm) after shaving the hair over the collection area. The skin sample is then soaked in saline solution for 10 min at 37°C or at room temperature overnight. The sediment is collected and examined under light microscopy as is or after adding a drop (50 μl) of methylene blue at 1% (46, 100) and observed under light microscopy at × 100 magnification.

Previous studies demonstrated that both *Cercopithifilaria* and *Onchocerca* microfilariae are unevenly distributed on the dog's body, with predominance in specific areas coinciding with vector feeding behavior for *C. bainae* (54). In dogs, microfilariae have higher frequencies on inter scapular region and on the head (54), known common (i.e., scapular region and head) sites of tick attachment. Hence, the predominance of microfilariae in these anatomical regions may be related to the long feeding period of ticks and consequently to *Cercopithifilaria* transmission (54). A similar study conducted with *O. lupi* positive dogs demonstrated a predominance of microfilariae on the nose and ears of dogs (100). Previously, the peri-ocular and umbilical areas were considered preferential sites for skin snipping (104). However, in this preceding research, the number of microfilariae was most likely underestimated as the skin snips were soaked for a short period of time (i.e., 1 h) (104), compared to up to 12 h in the more recent study (100). Data about predilection sites of microfilariae distribution and the time of skin soak described above are extremely useful to veterinarians since may increase the sensitivity of the technique employed. Nonetheless, considering that the collection of skin fragments is an invasive procedure, other factors such as practicability and vascularization of the anatomical region might be considered (100).

Morphometric analysis under microscopy is imperative for genus- or species-level identification of microfilariae recovered from skin snips as well as those circulating in the blood stream. Even if morphometric data of these microfilariae have been provided in previous research, these often do not consider some important aspects including age and origin of the material or detailed fixation or staining methods employed. Both blood and dermal microfilariae may be recovered through different techniques such as modified Knott's, Giemsa or hematoxylin staining of blood smears, saline sedimentation, impression smears, fine needle aspirates, or cytology spin preps. Additionally, reports of molecular detection of *C. bainae* microfilariae in dog blood (55, 56, 112), although likely introduced from the skin during venipuncture, highlight the importance of an accurate morphometrical analysis of circulating microfilariae to avoid misdiagnosis. Morphological features for all known blood and dermal microfilariae species infecting dogs have been summarized in Table 2 for modified Knott's and saline sedimentation and in Supplementary Table 1 for blood smears.

**Table 2:** Morphological and morphometrical data of microfilariae present in the blood and skin of dogs.

***Dirofilaria immitis***				
**Diagnostic method**	**Morphology**	**Range (**μ**m; mean** ±**SD)**		**References**
Modified Knott's test	Conical, tapered anterior, and straight tail	**Length**	**Width**	
		NR (301.7 ± 6.3)	NR (6.3 ± 0.3)	(11)
		292.9 – 339.8 (NR)	5.4 – 6.4 (6.2)	(113, 114)^‡^
		290.7 – 309.7 (302)	5.4 – 6.4 (6.2)	(115)
		284 – 303 (297)	5.9 – 7.1 (6.6)	(116)^‡^
***Dirofilaria repens***				
Modified Knott's test	Conical anterior, often curved caudal end or umbrella handled tail	NR (369.4 ± 10.8)	NR (8.87 ± 0.58)	(11)
		351.54 – 379.44 (NR)	7.05 – 8.3 (NR)	(115)
		342 – 392 (NR)	NR (NR)	(117)
***Acanthocheilonema reconditum***				
Modified Knott's test	Typically blunt anterior, often curved with or without button hooked tail	NR (254.4 ±7)	NR (4.63 ± 0.52)	(11)
		246.4 – 291.6 (70.9 ± 1.0)	4.7 – 5.8 (5.2 ± 0.02)	(113, 114)^‡^
		250 – 288 (263)	4.5 – 5.5 (NR)	(118)
		237 – 282 (259)	4.7 – 5.2 (NR)	(116)^‡^
***Acanthocheilonema dracunculoides***				
Modified Knott's test	Rounded anterior, straight tail, and internal body visible	NR (264.8 ± 5.5)	NR (5.1 ± 0.5)	(11)
		233 – 277 (NR)	4.5 – 6.0 (NR)	(119)^‡^
		213 – 265 (NR)	3.1 – 5.7 (NR)	(120)^‡^
		248.7 – 272.7 (NR)	5.1 – 6.7 (NR)	(121)^‡^
***Acanthocheilonema*** **sp**. ***“Ladhakii genotype”***				
Modified Knott's test	Sheathed	NR (320)	NR (NR)	(8, 9)
***Brugia pahangi***				
Modified Knott's test	NR	274 – 288 (NR)	5 – 6 (NR)	(122)^‡^
***Brugia patei*** **and** Brugiaceylonensis^*^				
	Sheathed	NR (NR)	NR (NR)	NA
***Brugia malayi***				
Modified Knott's test	Sheath, terminal and subterminal swelling	240 – 298 (NR)	NR (NR)	(123)
***Cercopithifilaria bainae***				
Saline sedimentation	Straight throughout and thick cuticle transverse striations	173 – 200 (NR)	5.6 – 7.5 (NR)	(40)
		182 – 190 (NR)	8.5 – 11 (NR)	(46)
***Cercopithifilaria*** **sp. II**				
Saline sedimentation	Conical anterior without alae and body with lateral alae	NR (287.9 ± 25.4)	NR (9.9 ± 1.3)	(124)
***Cercopithifilaria grassi***				
Saline sedimentation	Bulbous anterior and bifid tail	635 – 670 (NR)	15 – 17 (NR)	(48, 125)^‡^
***Onchocerca lupi***				
Saline sedimentation	Anterior bluntly rounded, straight, and bent tail	81 – 115 (NR)	4 – 6 (NR)	(97)^‡^

^*^Modified Knott test data not available.

^‡^Necropsy verified infections.

NR, Not Reported.

NA, Not Applicable.

**Figure 2:**
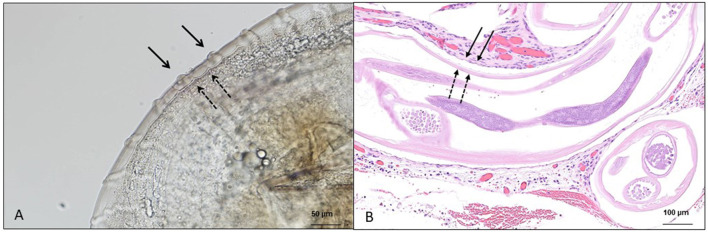
Microscopic features of *Onchocerca lupi* adults. **(A)** Multilayered cuticle with prominent annular ridges, external surface (full arrow), and internal layer (dashed arrow); scale bar: 50 μm. **(B)** Histological section of the ocular tissue of a dog with the presence of *O. lupi*, external surface (full arrow) and internal layer (dashed arrow); scale bar: 100 μm.

In addition to the research of microfilariae in skin snips, the retrieval of adult specimens (intact or degenerate) of *O. lupi* from ocular nodules has been a method of diagnosis that is obtained after surgical removal or biopsy of conjunctival tissue, followed by histopathological, morphological, and/or molecular identification (Figures 2A, B) (73, 126). At the histopathological examination, patterns of cuticular ridges of two inner striae within the space between two outer cuticular ridges have been observed and are morphologically compatible with *O. lupi* (73, 109). This procedure is invasive, and unfortunately, the diagnosis is achieved in animals after the presentation of clinical signs (73). Most often, these studies further confirm the morphological identification with DNA extraction, followed by different molecular tools (126).

## 3. Subcutaneous filarioid nematodes with blood microfilariae

### 3.1. *Acanthocheilonema* spp.

#### 3.1.1. Biology

The genus *Acanthocheilonema* has three species known to infect dogs, the most common being *Acanthocheilonema reconditum* (formerly *Dipetalonema reconditum*), *A. dracunculoides* (formerly *Dipetalonema dracunculoides*), and an undescribed species reported in the blood of dogs in Ladhak, northern India (8, 9). Adult nematodes of *A. reconditum* are present in the subcutaneous tissues of the trunk, hind limbs and fascial spaces (33, 114, 116, 127) and *A. dracunculoides* in subcutaneous tissues, peritoneal, thoracic, and abdominal cavities, or hind legs (120, 121, 128, 129). Of these, only *A. reconditum* has been reported from the United States.

Known arthropod vectors and development times to infective stage (L3) vary for the different canine *Acanthocheilonema* species. *Acanthocheilonema reconditum* is vectored by fleas, including *Ctenocephalides canis* and *Ctenocephalides felis*, and lice, *Heterodoxus spiniger* and *Linognathus setosus* (33, 127, 130). Development times in *C. felis* maintained on the infected host dog was 7–10 days (116, 131), whereas in off-host lab-maintained fleas fed an artificial bloodmeal, full development took 15 days (132). No development occurs in the brown dog tick, *R. sanguineus* (s.l) (132). The time of development for *A. dracunculoides* is unknown in the dog louse fly, *Hippobosca longipennis* (9, 129), and takes ~7 days in *R. sanguineus* (s.l.) nymphs (133). Interestingly, no developing forms were seen in the *R. sanguineus* (s.l.) ticks removed from a microfilaremic dog, which was found to be infected with adults at necropsy and co-infested by *H. longipennis* (129). In addition, it is unknown whether all members of the species complex of *R. sanguineus* (s.l.) ticks are equally competent vectors of *A*. *dracunculoides*, and this would require biological confirmation through experimental trials. Nevertheless, upon a potential introduction of *A. dracunculoides* to North America, *R. sanguineus* would be more likely to vector this species, as *H. longipennis* is not endemic to this region.

Infective larvae of *A. reconditum* have been experimentally transmitted *via* direct penetration of shaved skin and oral mucosa of anesthetized dogs (116, 131, 134). Whether transmission to the canine host occurs during vector blood feeding is uncertain, although it was noted by Pennington (116) that “infective larvae egressing from the intersclerical membranes around the mouth parts” and “infective larvae could be encouraged to come out” by manipulating the “mouthparts forward into a natural feeding position,” allowing escape through this membrane when stretched thin and finally allowing infective larvae to potentially penetrate the skin or bite wound (116). Ingestion of fleas was also suggested to be a more common route of infection (116, 132). It is plausible that multiple routes of infection in dogs may occur naturally.

**Figure 3:**
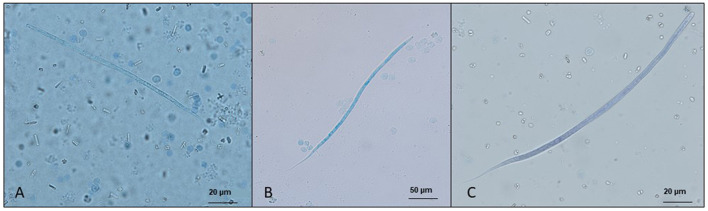
Microfilariae detected in blood samples by a modified Knott's test and stained with methylene blue. **(A)** *Acanthocheilonema reconditum*; scale bar: 20 μm. **(B)** *Dirofilaria immitis*; scale bar: 50 μm. **(C)** *Dirofilaria repens*; scale bar 20 μm.

Upon development and maturation in the subcutaneous tissues, gravid females release microfilariae into surrounding tissues and arrive in blood circulation 67–101-day post-infection for *A. reconditum* (Figures 3A–C), with a maximum patency period of 795 days (114, 116, 131, 134–136) and 69–76-day post-infection for *A. dracunculoides* (1, 131, 133, 136). Microfilaremia is often low in natural infections, ranging from 1 to 482 mf/ml of blood for *A. reconditum* to 5,050 mf/ml for *A*. *dracunculoides*. In a rare case, a dog from Spain with 791 adults recovered at necropsy, microfilaremia reached 264,367 mf/ml (121).

#### 3.1.2. Geographic distribution

While *A. reconditum* is considered globally distributed, *A. dracunculoides* is found in Europe (particularly Spain), Africa, and West Asia. The reported prevalence and distribution of both species are likely underestimated due to low numbers of microfilaremia and limitations of classical and molecular techniques, particularly in cases of co-infections with *Dirofilaria* species. Most surveillance for *A. reconditum* occurred prior to the adoption of the heartworm antigen tests in 1985, when clinical signs and identification of microfilariae were required for the diagnosis of heartworm. It has been reported in at least 22 states in the US (27, 137–139). Most likely, the distribution of *A. reconditum* is more widespread in the country; however, due to the lack of clinical relevance, cases routinely diagnosed by practitioners and diagnostic laboratories are not usually published. Epidemiological surveys and the prevalence of this parasite in Europe were recently summarized, reporting 64 surveys from 1987 to 2019 (140). In contrast, there have been scarce reports from the US during the same period (20, 21), which should underscore the need for more active surveillance using a combination of diagnostic methods, including classical (141), histochemical (142), and molecular methods (143). Despite the need for more active surveillance, the routine use of ectoparasiticide compounds observed in the last decades has likely contributed to the reduction of infection by *Acanthocheilonema* species in dogs.

#### 3.1.3. Pathogenicity, clinical signs, and diagnosis

While generally considered a non-pathogenic parasite (52, 131), *A. reconditum* experimentally infected dogs presented with eosinophilia, and in chronic infections, this abnormality was accompanied by proteinuria presumed to be due to microfilariae (131). Newly reported molecularly confirmed cases in Colombia among dogs testing positive for *A. reconditum* were statistically associated with male dogs showing clinical signs of anemia, including low levels of hemoglobin and hematocrit and elevated levels of plasma proteins (144).

Currently, *in vivo* diagnosis of *Acanthocheilonema* spp. is mainly achieved by morphological or molecular identification of the microfilariae in the blood (27, 55). Morphometric differentiation by a modified Knott's test between the two established canine species of *Acanthocheilonema* is not reliable and requires acid phosphatase staining or molecular differentiation (11, 131). However, with the current assumption that only *A. reconditum* occurs in the US, it is likely that a potential *A. dracunculoides* case would be misdiagnosed. The best techniques to detect low microfilaremia, excluding xenodiagnoses (127), include filtration methods (145) and molecular detection of DNA *via* conventional or qPCR of whole blood samples. When coinfections are present, low *Acanthocheilonema* microfilaremia may be overlooked among the numerous *Dirofilaria* microfilariae. This co-infection scenario may also explain the reduced sensitivity of some species-specific PCR assays in cases where the ratio of one species to another is high (24, 146, 147) or the inability to molecularly confirm co-infections of *A. reconditum* and *D. immitis* seen following examination of the entire Knott's sediment (24). More accurate diagnosis may be achieved with more sensitive molecular assays such as single or multiplex probe-based PCR protocols (27, 57, 148), accompanied by the sequencing of amplicons as an additional confirmation.

A range of morphologic characteristics, including length, width, the shape of the anterior and posterior extremities, and acid phosphatase somatic staining patterns, were described for microfilariae of *A. reconditum* (118, 149, 150). This morphological variation for *A. reconditum* was acknowledged by Courtney et al. (151) in natural populations throughout Florida and by Lindemann et al. (131), who observed more morphometric variability among naturally vs. experimentally infected dogs for diagnostic features such as the blunt head and curved buttonhook tail and found acid phosphatase necessary to determine an identity for microfilariae 270–290 μm in length. Some studies have suggested that these observations are evidence that more than a single *Acanthocheilonema* species are present within the US, with no additional investigations published (118, 149, 150). Although, these differences in morphology could represent intraspecific variability within *A. reconditum*, an alternative hypothesis would be that *A. dracunculoides* may be present in the US, with acid phosphatase staining described by Price (150), matching those described for this species (152, 153). Representative molecular sequences of *A. reconditum* from the US are currently lacking in Genbank, as are those of *A. dracunculoides* from other parts of the world. Informative morphological, histochemical, and molecular data from microfilariae surveys in dogs and vectors in the US are lacking. As isolates are obtained, molecular confirmation by multiple gene targets and the addition of the sequences to public repositories are needed. The only published investigation of filarial DNA in the known vector *R. sanguineus* (s.l.) submitted by veterinarians from pet dogs, identified only in *C. bainae*, also transmitted by these ticks (40).

## 4. Other filarioid nematodes with “potential” cutaneous localization

### 4.1. *Brugia* spp.

*Brugia* spp. are filarial nematodes that parasitize the lymphatic system of several mammal species, including dogs and humans (154). Microfilariae are released into the bloodstream and developed into mosquito vectors (Culicidae) (1, 155). The transmission through mosquitoes, which have a broader distribution worldwide, it may contribute for the potential spread of these parasites in areas where vertebrate susceptible hosts are present and mosquito vectors are abundant (156). Despite being composed of at least 10 species, *B. pahangi* and *B. malayi* are those more studied due to their occurrence in human populations, especially in Africa and Asia (156, 157). In dogs, the pathogenic importance of these infections has been considered minimal, but in some cases, lymphadenomegaly and lymphedema may be observed (158). Other canine species reported in dogs include *B. ceylonensis* in India and Sri Lanka, *B. patei* in Kenya (Pate Island), and *Brugia* species presenting high similarity to *B. malayi* and *B. pahangi* in Chad (159, 160).

In the US, the infection in wild animals for *Brugia beaveri*, infecting *Procyon lotor, Felis lynx*, and possibly *Neogale vison*, and for *Brugia lepori*, infecting lagomorphs such as *Sylvilagus aquaticus* and *Sylvilagus floridanus*, has been documented (161–166), but no case in dogs has been reported. Recently, in Canada, an unusual case of *Brugia* infection was noticed in a dog presenting with a subcutaneous mass (167). Even if not detected specifically in the US, this case sounds like an alert for veterinarians in North America who might consider the potential for infection in dogs with different *Brugia* species. Additionally, *Brugia* infection has been reported in a cat from California (162) and various humans across the US (and Canada) (168–171). The diagnosis of *Brugia* species has been mentioned in the section that covers the morphological differentiation of microfilariae in the blood and skin of dogs.

### 4.2. *Dirofilaria* spp.

The genus *Dirofilaria* is composed of at least 27 species infecting carnivores, insectivores, and marsupials worldwide (172), with at least three species of importance infecting dogs. Most species within the genus *Dirofilaria* primarily develop into adults and remain in the subcutaneous tissues except for *D. immitis* found in the cardiopulmonary vasculature. Additionally, all *Dirofilaria* species develop up to infective L3 stage generally over 14 days dependent on temperature and are vectored especially by *Culex* spp., *Aedes* spp., and *Anopheles* spp. mosquitoes worldwide except for *Dirofilaria ursi* infecting bears that are vectored by black flies (*Simulium* spp.) (18, 173, 174). The life cycle of *Dirofilaria* species is long, ranging from 6 to 9 months for *D. immitis* and from 6 to 8 months for *D. repens* and *Dirofilaria* sp. (“hongkongensis” genotype) following infection to when microfilaria is seen in the blood stream. Several *Dirofilaria* species are documented to be zoonotic, and recently, a patent human infection of *D. immitis* and *D. repens* was reported in asymptomatic individuals (175).

*Dirofilaria repens* is the primary species causing canine subcutaneous dirofilariosis, which is endemic in Europe, southeast Asia, and Africa (176, 177). It is primarily of zoonotic importance but can be highly prevalent in dogs (178). Similar to other representatives of *Dirofilaria*, it can be transmitted by numerous mosquito species and may establish in the same areas suitable for *D. immitis* due to the possibility of competent vector sharing (173). Additionally, it can be easily confused with *D. immitis* in geographic areas where endemicity is not recognized. Adults are commonly found under the skin in the fascia sheaths of muscles (179). Despite that most *D. repens* canine cases infections are asymptomatic, non-specific clinical signs such as pruritus, itching, and asthenia may be present (176). In addition, alopecic areas with hyperpigmentation and an uncommon case of diffused dermatitis associated with *D. repens* have also been described (180). The recent introduction of non-endemic vector-borne canine pathogens such as *D. repens*, reported in ~2% of both dogs and cats in South Florida, should not be surprising with requirements for animal importation only requiring vaccination records, a negative rabies titer, or origination from a low-risk country (17). It is important to highlight that no antigen test is available for *D. repens*, and the only way to reach a differential diagnosis is through the morphometric features of circulating microfilariae or by molecular tools.

*Dirofilaria immitis*, the causative agent of canine heartworm disease, is regarded as the most important parasite infecting dogs, has a global distribution, and is the primary filarioid of research focus and surveillance in the canine population, particularly in the US. Importantly, the full developmental life cycle in the vertebrate host has been known for a long time (181). While primarily found in the pulmonary arteries, right heart, and associated vasculature, there are increasing case reports and awareness of its presence in other subcutaneous, extravascular, and aberrant locations manifesting in diverse clinical presentations (182–186). Nematodes found incidentally outside the cardiopulmonary vasculature, in nodules, subcutaneous tissues, or body cavities should not be dismissed as heartworm and should be submitted to a veterinary parasitologist or veterinary reference lab for definitive identification. Due to the overreliance on heartworm antigen tests and a lack of microfilaria testing, heartworm prevalence remains underestimated, as recently highlighted in studies using immune complex dissociation (ICD) methods such as acid or heat treatment (20, 24, 186). The pathogenicity of *D. immitis* infections is a consequence of the anatomical localization of adult parasites as well as the presence of circulating microfilariae (18). The most common clinical presentation is chronic, characterized by dyspnea, weakness, anorexia, and ascites, which may be triggered as a consequence of heart failure (18). Because the pathogenicity of *D. immitis* infections has been extensively studied, the clinical signs of this parasite will not be discussed in detail here since numerous published resources are available for reference (18).

There is evidence of cryptic diversity among what was thought to be *D. repens* circulating in dogs (10), causing human cases in south India and Hong Kong (10, 15, 187–189). For example, molecularly, *D. repens* microfilariae are similar to those of a newly recognized *Dirofilaria* sp. (“hongkongensis” genotype), with the 18s molecular gene target unable to differentiate the two species, thus appropriate molecular targets should be emphasized (188). Morphological descriptions of the adult worms and microfilariae are lacking for this putative species.

It is not clear if *D. repens* cross-reacts with the global array of heartworm antigen tests, but it is known to cross-react with a commercial heartworm antigen test following immune complex dissociation (ICD) *via* heat treatment (107). Differently, *A. reconditum* and *O. lupi* do not seem to cross-react with a few commercial heartworm antigen-detection kits that have been tested following this ICD protocol (24, 27, 107).

## 5. Morphological identification of microfilariae in blood and skin

Most microfilariae testing is focused on detecting the canine heartworm and differentiating it from other globally recognized species with blood microfilariae (Table 2 and Supplementary Table 1). The modified Knott's method (141) is the most commonly used method for morphometric differentiation of unsheathed blood microfilariae based on morphological characteristics (11). While the morphological characteristics of a modified Knott's preparation are preserved over time (11, 190), the time from collection to processing can apparently cause morphology differences within a sample from the same dog (135).

The use of thin and thick blood smears stained by Giemsa or hematoxylin, an alternative differentiation method, is often used in regions where sheathed microfilariae of *Brugia* species are common together with unsheathed microfilariae. The length of the cephalic space and innenkorper are considered major differentiating morphological features for *Brugia* species, which also have two distinct and separate nuclei in the tail differentiating this genus from *Wuchereria bancrofti* in humans (161, 191, 192). In fixed blood smears, *D. immitis* can be clearly distinguished from *D. repens D. striata*, and “*D. striata-like*” which have been rarely reported in dogs from Florida. These microfilariae can easily be distinguished from *D. immitis* due to the presence of two nuclei distinctly separate from the nuclear column in cephalic space in these latter species (118, 193). Variation in reported length measurements for morphological features observed in blood smears can be caused by the numerous different solvents used for fixing dried blood smears, which causes different degrees of shrinkage of microfilariae. This variability of measurements in the literature for thin and thick smears is generally addressed by comparing percentages between reports, which is possible considering the length of each morphological feature from the anterior end divided by the microfilariae length (194). However, the lack of reported range measurements for each species and the use of different fixatives in the literature potentially complicate an accurate differentiation between species.

Unfortunately, a broad range of morphological measurements exists for the modified Knott's and stained thin or thick blood smears, and measurements from these different methods are often lumped together in reference tables causing some confusion. Clarification of the literature should be considered using results from multiple diagnostic tools (Knotts, Giemsa thin blood and thick blood smears, acid phosphatase, and PCR of multiple gene targets), in a similar fashion recently used to demonstrate *B. malayi* is indeed infecting dogs in southern India and possibly impacting eradication efforts (14). We have attempted to clarify the reported morphological measurements between the species for modified Knott's with the inclusion of saline sedimentation in Table 2. For techniques using blood smears, consistent reporting of morphological characters or the lack of details on fixation and staining methods in the literature that hindered our efforts, further clarification identifying microfilariae in blood smears may be necessary. Available morphological information using this technique, for which methodology was discernible or where no other data sources were available, is summarized in Supplementary Table 1.

**Figure 4:**
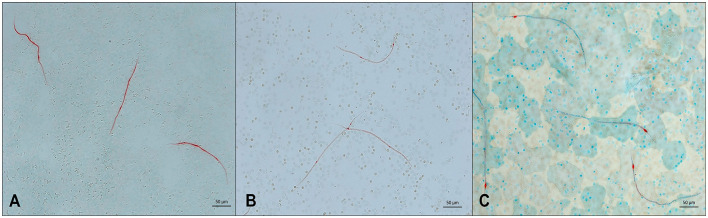
Microfilariae detected in blood samples stained by acid phosphatase (AP) histochemical method. **(A)** *Acantocheilonema reconditum:* AP activity through the entire body (red); scale bar: 50 μm. **(B)** *Dirofilaria immitis*: AP activity in excretory and anal pores (red); scale bar: 50 μm. **(C)** *Dirofilaria repens*: AP activity in anal pores (red); scale bar: 50 μm.

Acid phosphatase staining is a particularly useful tool for differentiating canine microfilariae (Figures 4A–C) (192), although laborious and time-consuming, thus generally restricted to diagnostic labs (11). Although the use of several commercially available kits has been described in the literature, these methods do not seem to work for all species of microfilariae (11, 195). The originally described method produces the most consistent results and is best used on freshly collected samples (142); however, the inclusion of a controlled drug in the veronal buffer hinders its accessible use. Reagents prepared for this technique are recommended to be used fresh but can also be combined as described (142), rapidly aliquoted, and flash frozen for longer shelf life and convenience (131). Using this staining method, *D. immitis* shows somatic staining at the excretory vesical and anal pore; *A. reconditum* shows diffuse staining throughout concentrated in the caudal half; *A. dracunculoides* demonstrates staining at the cephalic vesicle, excretory pore, inner body, and anal pore; and *D. repens* apparently has two different somatic staining patterns reported in the literature, specifically at the inner body and anal pore or the anal pore only (11, 13, 20, 142). Whether these somatic staining patterns are different between *D. repens* and the *Dirofilaria* sp., “hongkongensis genotype” is unknown but should be confirmed along with specific molecular targets along with other identified genotypes possibly distinct from *D. repens*. Somatic staining patterns for *Brugia* spp. infecting dogs have been previously discussed elsewhere (13, 14, 159).

## 6. Molecular differentiation of microfilariae in blood and skin

Due to the limitations presented by microscopy-based techniques, molecular tools have been extensively used for the diagnosis of filarioid in dogs. These DNA-based techniques provide important genetic data useful for the differentiation and detection of mixed infections (55). Initially employed for blood microfilariae (e.g., *Dirofilaria* sp. and *Acanthocheilonema* sp.), these methods allowed specific diagnosis in vertebrate and invertebrate hosts (196, 197) or differentiation between *Brugia* spp. (160, 198). However, in endemic regions, where more than one filarioid species is detected, the differentiation of these parasites is pivotal (199, 200).

It is indisputable the advances of DNA-based tests over the last years, but in general, these methods were able to differentiate a few filarioid species present in the blood stream (197). With the retrieval of subcutaneous species (*Cercopithifilaria* sp. and *O. lupi*) infecting dogs, the molecular methods focusing only on blood parasites (30) became outdated in regions where subcutaneous species were also detected (31, 73). Then, molecular techniques capable of detecting three or more filarioids simultaneously were developed. For example, in 2012, a multiplex PCR for the simultaneous detection and differentiation of *Dirofilaria* sp., *A. reconditum*, and *Cercopithifilaria* sp. was proposed (201). Afterward, a HRM real-time qPCR was successfully developed for the quantification of *D. immitis*-microfilariae and distinguished filarioid nematodes that were not detected by the other employed assays (55), being a good tool for the detection of samples with low microfilarial concentrations likely missed by the modified Knott's test (145). More recently, a multiplex real-time PCR allowed specific detection of bloodstream filarioid nematodes, in which the authors suggest that this technique can detect occult infection by *Dirofilaria* spp. when interpreted together with results from blood smears and heartworm antigen testing (148). Additional studies are needed to support claims of molecular detection of occult infection and evaluated against filtration microfilariae testing, necropsy, and recovery of nematodes from tissues (24). Apart from filarial parasites, this tool also targets *Wolbachia* genotypes, an application important for diagnosis and also follow-up on animals undergoing treatment (148, 202).

The rise in cases of *O. lupi* infecting dogs and humans in Europe and North America has stimulated the development of novel molecular diagnostic protocols aiming for higher sensitivity and specificity. It is important to note that microfilariae of this species are skin-dwelling (100), and these are likely to go undetected or underdiagnosed as the collection and screening of skin samples are less common than those of blood samples. The development of the molecular diagnosis of cutaneous filarioid species in dogs has expanded given the increased interest in these parasites. In most cases, it is based on the conventional PCR approach followed by sequencing, which is labor- and time-intensive and unpractical for large-scale epidemiological studies. Then, a species-specific qPCR assay developed represents an important resource for the diagnosis of *O*. *lupi* and for the detection and quantification of the low number of microfilariae of this nematode (85).

Recently, the mitogenome of *O. lupi* was provided through the analysis of next-generation sequencing and molecular phylogenetic placement, which revealed that it is composed of 13,766 bp and contains 36 genes (203). These data are useful for studies involving different aspects of this filarioid, including the discovery of new markers important for the development of diagnostic tools. Stimulated by this finding of the mitogenome, a real-time PCR was developed to determine host-to-parasite DNA ratios (*Onchocerca lupi* vs. *Canis lupus*), which are essential for knowledge about the whole genome sequence (204), but can also be very important for future diagnostic purposes.

Despite the advances in molecular diagnostics, these tools have only recently been applied for the screening of filarioid species in pet and shelter populations in the US, sometimes in combination with morphological identifications (20, 23–27, 32). Additionally, efforts should be made to design molecular assays that can overcome a dominant species bias, as seen for *D. immitis/D. repens* coinfections (147) and *D. immitis/A. reconditum* coinfections (24, 146).

## 7. Reports of other microfilariae of unknown identity

Increased surveillance in pet and shelter canine populations may occasionally result in the detection of microfilariae that do not fit morphologically with known species (Table 3). In the Americas, microfilariae of diverse morphometry and unknown identity have been sporadically detected in canine skin, tissues, lesions, and blood but are lacking further investigation and description. For example, in Florida, *Dirofilaria striata*, of wild felids, or *D. striata*-like microfilariae was reported in 3 dogs (151, 206) during modified Knott's testing, and larger microfilariae similar in size to *D. lutrae*, which infects otters (199), was reported in 1 dog with only adults of *D. immitis* at necropsy (24). Only dermal microfilariae were recovered in 10 dogs from Arizona, with dermal lesions (e.g., papules, alopecia, scarring, erythema, ulceration, and crusting) attributed to *Acanthocheilonema* species based on the recovery of 1 adult female, which emerged from an excised nodule (192). Also, a single dermal microfilaria from the skin of a dog in Oklahoma, shorter than that of *C. bainae*, were reported in a recent study but could not be molecularly characterized (40). Additionally, in Brazil, microfilariae measuring ~168 μm in length were recovered from skin snips and detected through histopathological examination in corneal biopsies of 8 dogs with ocular keratitis (208).

**Table 3:** Microfilariae that do not fit morphologically with known species of occurrence in the Americas.

**Location of infection (*n*)**	**Clinical signs (findings)**	**Morphology (measure μm)**	**Assumed species**	**References**
Florida—US (1)	ND (microfilariae in blood)	Nerve ring (72), two nuclei separate from nuclear column in cephalic space (368 ± 3 length and 6 width)	*Dirofilaria striata*	(190)
Florida—US (2)	ND (microfilariae in blood)	Tapered anterior, variable curved tail. 2 nuclei separate from nuclear column in cephalic space (360 – 385 length and 5 – 6 width)	*D. striata*-like “Florida *Dirofilaria* sp.”	(118, 151)
Florida—US (1)	ND (2 microfilariae in blood among *D. immitis ~*30, 000 mf/ml)	Tapered head, curved body and tail (1. 427 length and 7.6 width; 2. 408.6 length and 7.3 width)	*D. lutrae*	(24, 205, 206)
Oklahoma—US (1)	ND (microfilariae in skin)	ND (160 length and 4.5 width)	NI	(40)
New Mexico, Arizona, Colorado, and Washington—US (10)	Papules with alopecia, scarring, erythema, ulceration, crusting or lesional pruritus (microfilariae in skin, 1 female adult emerged from biopsy)	ND	*Acanthocheilonema* sp.	(199)
Canada (1)	Facial subcutaneous nodule	NA	*Brugia* sp.	(167)
Brazil (8)	Superficial corneal opacities, bilateral lesions associated with mild to moderate conjunctival hyperemia. Dermal lesions from head neck, interscapular, and lumbar regions (ocular microfilaria in corneal stroma and in saline from skin snips)	Straight and unsheathed, tapered anterior rounded, pointed tail (168 length and 5 width)	*Onchocerca* sp. or *Cercopithifilaria* sp.	(207)

NA, Not Applicable; ND, Nor Described; NI, Not Indicated.

Apart from dogs, large microfilariae of unknown morphological and molecular identity were observed in a cat from Florida and in a bobcat from Oklahoma. In both cases, gene sequences differed from those published for *D. striata* (23, 25). Modified Knott's testing of blood from Florida panthers reported diverse morphology and a wide range of lengths (273–370 μm), many smaller than expected for *D. striata* (195). Although those microfilariae were found in felids, these examples should emphasize our lack of understanding of filarioid nematodes in domestic and wild animals in the US. When possible, microfilariae of unknown identity or subcutaneous nodules should be investigated with multiple techniques, which could possibly aid in the identification and, ideally, recovery of adult specimens on the occasion of necroscopic examination. When a dog or cat is microfilariae-positive and heartworm antigen-negative, it is strongly encouraged that the sample should be examined in a reference diagnostic laboratory (20), ideally with the capability for molecularly characterizing the sample.

## 8. Zoonosis and proactive surveillance for filarioids in domestic and wild animals

Nematodes of the genera *Brugia* and *Dirofilaria* present in mammals pose the greatest zoonotic risk due to their ability to infect and adapt to a wide range of hosts (45, 209). All species of canine filarioids should be generally suspected of zoonotic potential due to the close association of canids with human communities (8). To date, the seven species that have been implicated in known human cases are *A. reconditum, B. pahangi, B. ceylonensis, D. immitis, D. repens, Dirofilaria* sp. (*hongkongensis genotype*), and *O. lupi* (10, 34, 45, 203, 210, 211). An increased risk of zoonosis, or anthropozoonosis for *B. malayi*, exists in areas where competent vectors also feed on both humans and infected canid/felid definitive hosts (212).

Surveillance activities, while specifically of veterinary and scientific interest, should also serve a public health interest, integrating into a one-health approach. An example of this approach would be to work with the state's health departments or existing mosquito control districts' arbovirus surveillance efforts to retest nucleic acid from mosquito pools and for the possible use of biting midges and black fly bycatch, while prioritizing geographic areas near identified microfilaremic animals. Alternatively, banked vector lysates from blue tongue, or other animal disease surveillance could also be repurposed. Additionally, population control activities and conservation activities by the state's wildlife departments may offer collaboration opportunities for use of banked samples or collection of carcasses, for skin, blood, tissue, or nematode recovery from wild canids, felids, and other mammals. Trap-neuter-release efforts for feral and free-roaming cats across the US provide an opportunity for blood collection and result in discarded ear tips, indicating vaccination, which could be opportunistically saved and used to screen for microfilariae of filarioids and other vector-borne diseases in cats. Unfortunately, impactful surveillance for these nematodes in arthropod vectors, wildlife, or domesticated hosts is often motivated in reaction to human cases.

Humans are considered “dead end” hosts for *D. immitis* and for *D. repens*, which are causative agents of cardiopulmonary and subcutaneous nodules, respectively (18). This established perspective may require additional examination with patent infections of *Dirofilaria* spp. detected molecularly in ~1% of asymptomatic healthy individuals in Italy (3/397 and 1/397 for *D. immitis* and *D. repens*, respectively) (175), and serologically in 6.1% (41/668 for *D. immitis* and *Wolbachia* surface protein antibodies) of patients in Portugal (213). Similar investigative studies could be performed on opportunistic blood samples from human populations from across the US, and positive results may spur a public health interest in control efforts targeting these parasites in dog and cat populations (i.e., heartworm prophylaxis). However, subcutaneous dirofilariosis caused by *D. repens* is the fastest growing zoonosis in Europe (214) in North America subcutaneous dirofilariosis caused by species associated with wildlife has so far only been suggested for *D. tenuis, D. ursi*, and *D. striata* (36, 209, 215–218).

The absence of a confirmed vector for *O. lupi* poses an additional impairment for the control and prevention of this infection in animal and human hosts. However, it is essential the implementation of surveillance activities due to the rise of human cases recently observed in the US (39). After the first case reported in Arizona (36), other states such as New Mexico and Texas diagnosed human patients, totaling 7 notifications in this country, most of them affecting children (6/7) (37–39). Along with the data on natural infection in dogs, the states of New Mexico and Arizona show up as important endemic areas, and the surveillance in these areas is pivotal to better understand the real epidemiological situation of this filarial infection and consequently prevent future cases. The detection of microfilariae of *O. lupi* is achieved through the examination of skin snips (100) that are less frequently collected than blood samples used for the detection of other filarioid species. Even if not collected routinely, skin fragments are usually obtained by vet dermatologists for diagnostic purposes. These samples might be extremely useful for the surveillance of this infection, especially for the detection of asymptomatic dogs. Currently, the most important source of data about *O. lupi* infection in endemic regions is veterinary ophthalmologists, since the ocular manifestations are the most common clinical signs (71, 73). To better understand the epidemiology of *O. lupi* in endemic regions, dogs and other potential hosts should be enrolled and screened despite the presence of clinical ocular disease.

In addition to all the surveillance actions herein reported, the control of filarial infection in dogs is pivotal to preventing human cases. For instance, chemoprophylaxis through the use of lactones macrocyclic (e.g., ivermectin, selamectin, milbemycin oxime, and moxidectin) is recommended in areas of high risk of infection by *Dirofilaria* spp. due to the microfilaricidal action of these substances (18). Also, the prevention of vector-borne infestations by ticks, fleas, and mosquitoes through the use of repellent and ectoparasiticides compounds has been stimulated everywhere to prevent infection by filarioids and other vector-borne pathogens (26).

## 9. Gaps in knowledge and future perspectives

The implementation of active surveillance for filarioid nematode infections and co-infections in dogs is necessary. Ideally, this effort should be geographically broad and utilize diagnostic methods that may detect endemic, emerging, and exotic species. Integrated morphological, robust molecular (e.g., multiple genes, mitogenomes, or whole genomes), and histochemical characterization of filarioid nematodes infecting wild animals and their vectors is warranted to better understand filarioid (cryptic) biodiversity, their host range, and geographic distribution (219). Geographic areas where microfilaremic imported animals are identified should be prioritized for surveillance in dogs, cats, and potential vectors. Previously uncharacterized species may occasionally infect dogs, such as the recent *Brugia* case in Canada, and also cats. These (un)known nematodes may represent a risk for both companion animals and humans, as a great proportion of zoonotic diseases come from wildlife reservoir species (220, 221).

Molecular characterization of all known *Dirofilaria* species in the New World is still lacking, despite recently added limited data for *D. lutrae* (207), *D. ursi* (221), and *D. striata* (222). For instance, recent studies focused on North American *Onchocerca* species have revealed various cryptic genetic lineages (223–227). Addressing this need may be important for identifying filarioid species in the future domestic animal and zoonotic cases (207, 228–234) or revisiting archival materials in museum collections that contain formalin-fixed tissues attributed to filarioid nematodes but that have not been unequivocally assigned to species. Serological surveillance for determining exposure or molecular screening for active infection by filarioids in human populations has not yet been examined in the US and represents a major knowledge gap (143).

In addition, some biological aspects of *O. lupi* and *C. bainae* life cycle and effective chemoprophylaxis and treatment and reliable diagnostic tools for the detection of infections and co-infections caused by filarioid nematodes occurring in sympatry may be considered important gaps in the knowledge of subcutaneous filarioid nematodes infecting dogs in the US. The research of new molecular markers and techniques, the training of parasitologists for classical microscopical examination, and a broad active surveillance program may improve parasite detection and inform veterinary and public health authorities for implementing intervention strategies for mitigating range expansion and the establishment of endemic and exotic filarioid species in animals and humans. With the exception of *D. immitis*, all of the filarioid nematode species discussed herein may be considered emerging, neglected, or underdiagnosed.

## Author contributions

All authors listed have made a substantial, direct, and intellectual contribution to the work and approved it for publication.
