# Supplementary material for: Cutaneous filarioid nematodes of dogs in the United States: Are they emerging, neglected, or underdiagnosed parasites?
Source: Front Vet Sci. 2023 Feb 23;10:1128611. doi: 10.3389/fvets.2023.1128611 (PMC9995907; doi:10.3389/fvets.2023.1128611)
Supplement: Supplementary file 1 [file Table_1.DOCX]

| **Supplementary 1 – Table.** Reported morphology (µm and/or % of total length) for blood smears for microfilariae of canine filarioids seen worldwide. | | | | | | | | | | | | | | |
| --- | --- | --- | --- | --- | --- | --- | --- | --- | --- | --- | --- | --- | --- | --- |
| **Species** | ***D. immitis*** | | ***D. repens*** | | ***A. reconditum*** | ***A. dracunculoides*** | ***B. pahangi*** | | ***B. patei*** | ***B. ceylonensis*** | ***B. malayi*** | | | ***B. beaveri*** |
| **Reference** | (124) | (33) | (124) | (125) | (33) | (125) | (120) | (125) | (126) | (127, 128) | (14) | (129) | (125) | (130) |
| **Fixative/Stain** | Methanol/ Giemsa | Giemsa | Methanol/ Giemsa |  | Giemsa |  | (60°C) 70% Ethanol/  Hematoxylin |  |  |  |  | (60°C) 70% Ethanol/  Hemalum |  | (60°C) 70% Ethanol/  Hematoxylin |
| **Sheath** | Absent | | | | | | Present | | | | | | | |
| **Total Length** | 232±6.35-292±8.4 |  | 311±27-332±4.9 | 290±10 |  | 195-230 | 186-200 |  | 180-230 | 220-275 | 190-220 | 177-230 | 220±20 | 200-258 |
| **First Nucleus (Width)** |  |  |  |  |  |  | 4 |  |  | 6-8 | 4.35-6.5 | 5.4-6.2 |  | 4.5-5.5 |
| **Cephalic space (Length)** | 3.13-11.38 |  | 1.04-8.18 |  |  |  | 10 (5.3%) |  | 4.8 | 6.3-6.7 | 8.6-10 | 6.9 |  | 11-14 (5.5%) |
| **Nerve Ring** | 55.4±4.1 | 23.8% | 66±4.3 | 23.0% | 20.8% | 45 | 42 (22.0%) | 22.0% |  | 30-73 | 45.35-48.5 |  | 24.5% | 48-70 (24%) |
| **Excretory Pore** |  | 32.7% |  | 30.0% | 21.0% | 66-70 | 58 (31.0%) | 31.0% |  | 65-100 | 61.88-78.5 | 30% | 35.0% | 68-90 (33%) |
| **Excretory cell** |  | 38.6% |  | 33.0% | 34.5% |  |  | 33.0% |  |  |  | 37% | 40.0% |  |
| **Innenkorper** |  |  |  |  |  |  | 92 (49.0%) |  |  |  |  | 40% |  | 110-145 (53%) |
| **Innenkorper end** |  |  |  |  |  |  | 131 (69.0%) |  |  |  |  | Near G1 |  | 147-185 (71%) |
| **G1 or R1 cell** |  | 67.9% |  | 62.5% | 70.1% |  | 135 (71.0%) | 68.0% |  |  |  | 68-72% | 64.0% | 150-188 (72%) |
| **G2 or R2 cell** |  | 74.1% |  |  | 75.7% |  |  | 73.0% |  |  |  | 70-77% |  |  |
| **G3 or R3 cell** |  | 75.4% |  |  | 77.2% |  |  | 74.0% |  |  |  | 72-78% |  |  |
| **G4 or R4 cell** |  | 79.4% |  | 66.0% | 78.6% |  | 168 (84.0%) | 78.0% |  |  |  | 74-81% | 80.0% | 160-213 (82%) |
| **Anal Pore** |  | 82.0% |  | 70.0% | 80.7% |  |  | 80.0% |  |  | 171.88-184.5 |  | 83.0% |  |
| **Last Nucleus** | 237.7±26.3 |  | 293±19.4 |  |  |  |  |  |  |  |  |  |  |  |
| **Last Tail Cell** |  | 92.9% |  |  | 89.0% | 53-55* |  |  |  |  |  |  |  |  |
| **Length of nucleus-free tail*** | 21.5±5.6* |  | 29.5±7.7* |  |  | 20* |  |  |  | 35* |  | 91-96%* |  |  |
| *Measured from posterior end | | | | | | | | | | | | | | |
